# Pancreatitis with an unusual fatal complication following endoscopic retrograde cholangiopancreaticography: a case report

**DOI:** 10.1186/1752-1947-2-215

**Published:** 2008-06-24

**Authors:** Boris Kanen, Ruud JLF Loffeld

**Affiliations:** 1 Department of Internal Medicine, Zaans Medisch Centrum, Zaandam, The Netherlands

## Abstract

**Introduction:**

Endoscopic retrograde cholangiopancreaticography has been the treatment of choice for stones in the common bile duct. Although the procedure is usually safe, procedure-related complications do occur.

**Case presentation:**

A case of pancreatitis following endoscopic retrograde cholangiopancreaticography is described in a 55-year-old woman. After an uneventful recovery the patient's condition deteriorated rapidly 16 days after the endoscopic retrograde cholangiopancreaticography, and the patient died within 1 hour. Post-mortem examination revealed massive intrapulmonary fat embolism. The complications of endoscopic retrograde cholangiopancreaticography and pancreatitis are described.

**Conclusion:**

Fat embolism can occur after the remission of pancreatitis and pancreatic necrosis may be overlooked on contrast-enhanced computed tomography scanning.

## Introduction

Endoscopic retrograde cholangiopancreaticography (ERCP) has been the treatment of choice for stones in the common bile duct. Although the procedure is usually safe, procedure-related complications do occur, the most serious of which are perforation, bleeding and pancreatitis. Pancreatitis can take a complicated course. Necrotising pancreatitis, pseudocysts, pancreatogenic ascites and infection have been reported. Systemic complications leading to multi-organ failure are the usual cause of death in cases of pancreatitis. However, post-ERCP pancreatitis is usually mild and self-limiting.

A patient with post-ERCP pancreatitis is described. During reconvalescence the patient developed a very rare secondary complication related to pancreatitis.

## Case presentation

A 55-year-old woman visited our clinic because of typical biliary colic. She had undergone cholecystectomy because of symptomatic gallstone disease 3 years earlier. For the last 6 months she had suffered from intermittently occurring colic. The pain was located in the right upper quadrant of the abdomen. The patient identified the complaint as being the same pain as she had experienced prior to the cholecystectomy. The colic was triggered by ingestion of fat. The patient also noted short periods of discoloured stools and dark urine without jaundice. In addition, the patient had classical reflux complaints with heartburn and acid regurgitation.

Laboratory investigations did not show any signs of cholestasis: aspartate aminotransferase (ASAT) 15 U/l (normal value 10 to 40 U/l), alanine aminotransferase (ALAT) 17 U/l (normal value 5 to 45 U/l), alkaline phosphatase 59 U/l (normal value 40 to 120 U/l) and bilirubin 9 μmol/l (normal value 1 to 15 μmol/l). However, an ultrasound investigation of the upper abdomen showed a slightly dilated common bile duct of 8 mm, with signs of small stones. Owing to the reflux complaints, an upper gastrointestinal endoscopy was performed. A hiatus hernia with reflux oesophagitis grade III according to Savary and Miller was diagnosed. It was decided that the patient should be treated with pantoprazol 40 mg daily. An ERCP was performed 4 weeks later. During this period there were no changes in the clinical condition with the exception of complete remission of the reflux complaints. A normal major papilla was seen. The pancreatic duct was normal. Despite several attempts it was not possible to gain access to the common bile duct. As the common bile duct was dilated it was decided to perform a precut papillotomy with the needle knife. Despite several attempts the common bile duct could not be cannulated and at that point the procedure was terminated.

Several hours later the patient complained of increasing pain in the upper part of her abdomen. The abdomen was tender with an absence of peristalsis. Examination of the blood revealed a serum amylase of 1142 U/l (normal value 60 to 220 U/l), ASAT of 1142 U/l (normal value 10 to 40 U/l), ALAT of 1220 U/l (normal value 5 to 45 U/l), alkaline phosphatase of 131 U/l (normal value 40 to 120 U/l) and γGT of 392 U/l (normal value 5 to 35 U/l). There was a leukocytosis of 15.6 × 10^9^/litre. An X-ray of the abdomen showed air in the retroperitoneal space. The clinical diagnosis was post-ERCP pancreatitis with perforation due to the precut papillotomy.

Computed tomography (CT) scanning with contrast enhancement 2 days later showed a right-sided pleural effusion and a collection of air in the retroperitoneal space. The head and corpus of the pancreas were normal; some infiltration in the region of the tail of the pancreas was seen. There was no necrosis. The common bile duct was dilated with a diameter of 1 cm.

The liver enzymes and hyperamylasaemia returned to normal within 3 days. On account of persisting leukocytosis and a body temperature of 38.3°C antibiotic therapy was started (cefuroxim and metronidazole). Blood cultures were negative. Enteral feeding via a tube in the proximal jejunum was started. Eight days after the onset of the pancreatitis the patient again developed fever (39.5°C) without obvious explanation. Blood investigations still showed a leukocytosis (17.3 × 10^9^/ml), with an acute phase reaction (elevation of the erythrocyte sedimentation rate and C-reactive protein). This time blood culture was positive for *Pseudomonas aeruginosa*. Antibiotic therapy was changed to ciprofloxacin. Her body temperature became subfebrile.

A CT scan taken 7 days later showed normalisation of the pancreas. The infiltration in the tail of the pancreas had almost subsided and the retroperitoneal air had disappeared. There was still pleural effusion and some ascites present. The clinical condition of the patient further improved, the fever disappeared and the abdomen was non-tender. The patient had normal stools. The treatment with opioids was tapered and normal oral feeding was started.

Sixteen days after the ERCP, when discharge of the patient was already being considered, her clinical condition deteriorated acutely. The patient became dyspnoeic, anxious and tachycardic, with a drop in blood pressure. Blood gas analysis showed respiratory alkalosis with hypoxaemia. Acute pulmonary embolism was suspected and treatment with anticoagulant therapy was started. Electrocardiography showed tachycardia with no signs of acute embolism or myocardial infarction. Cardiac ultrasound did not show signs of infarction or high pressures in the right side of the heart. The clinical situation worsened over the next 30 minutes. Ventricular tachycardia developed, respiratory arrest occurred and despite resuscitation the patient died within 1 hour.

Post-mortem examination showed no signs of acute myocardial infarction or pulmonary embolism. Both lungs showed signs of congestion compatible with the resuscitation. There was pleural fluid present. The culture was sterile. The pancreas showed signs of necrosis in the head and tail. There was fat necrosis in the retroperitoneum. No abscesses were seen. There were no signs of recent local bleeding. The common bile duct was dilated but there were no signs of stones in the common bile duct. A perforation opening at the level of the papilla was not detected. Two litres of ascites were present. There were no signs of systemic septicaemia, despite the fact that culture of the intra-abdominal fluid was positive for *Enterococcus faecalis*. Macroscopic examination was unable to identify any direct cause of death. Revision of the final CT scan did not reveal signs of necrosis.

**Figure 1:**
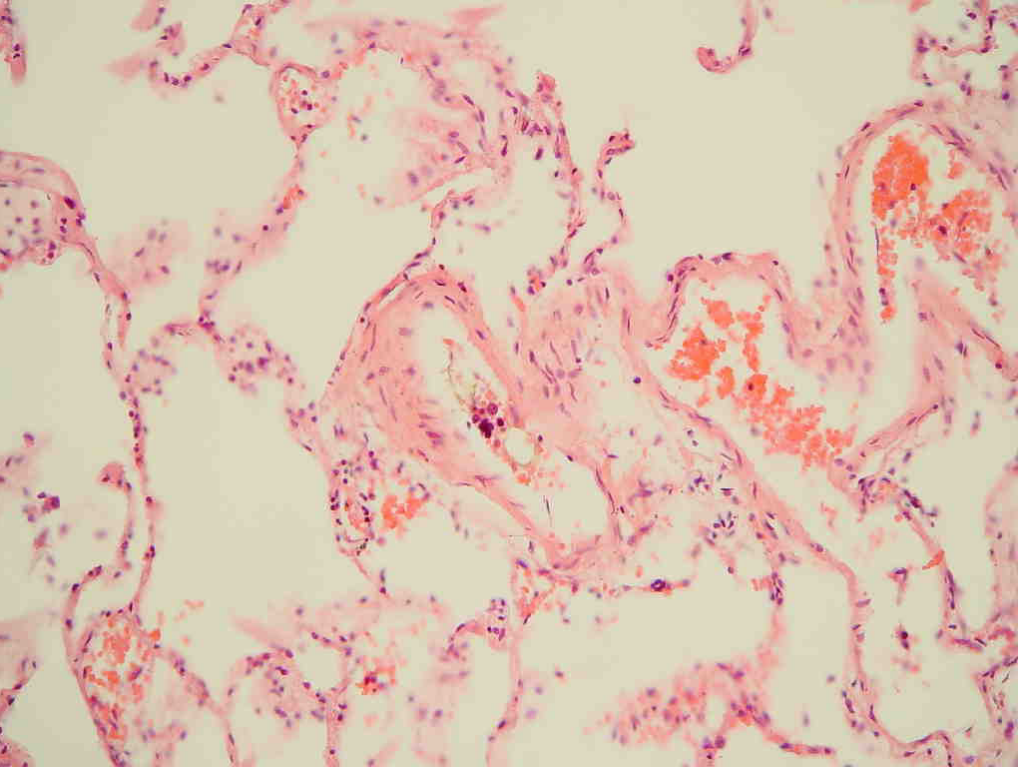
Haematoxylin and eosin stain of a section of the lungs showing a blood vessel with fibrinoid material and an optical empty space indicative of the presence of lipid dissolved during the staining process.

**Figure 2:**
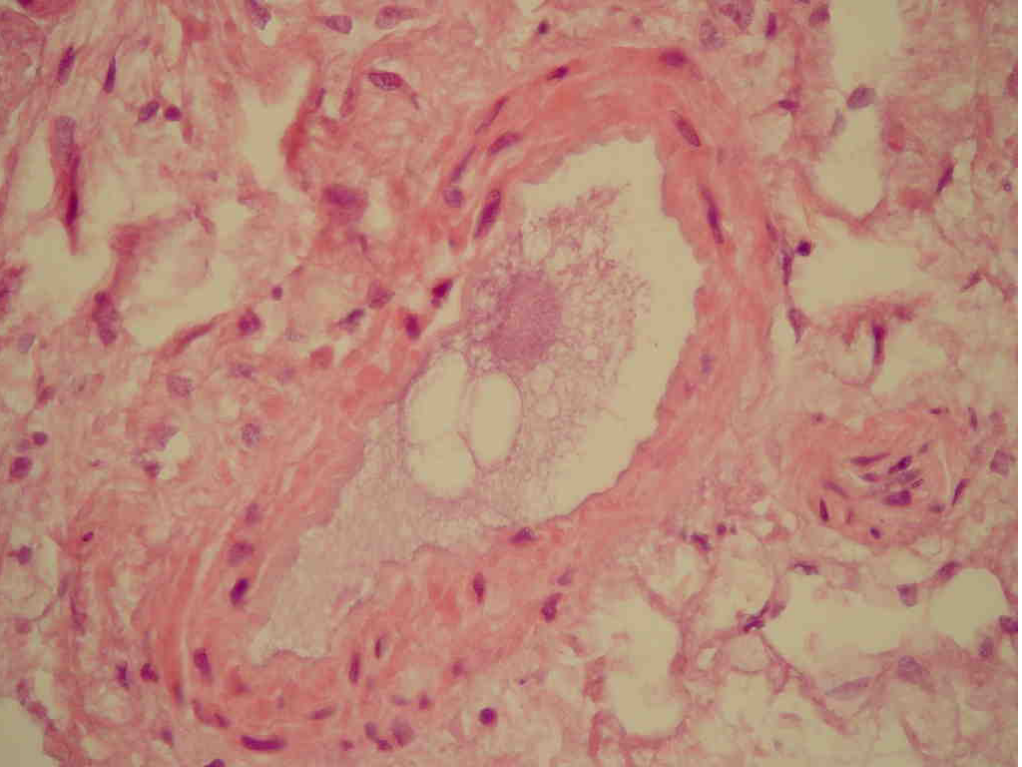
The phenomenon shown in Figure 1 at a higher magnification.

Histological examination of the pancreas showed normal pancreatic tissue as well as areas with fatty necrosis. However, histological examination of sections of the lungs showed signs consistent with massive intrapulmonary fat embolism (Figures 1 and 2). The final diagnosis was death due to massive intrapulmonary fat embolism which occurred 16 days after the onset of a post-ERCP pancreatitis.

## Discussion

This case report clearly highlights two important issues: first, the potential risk of ERCP, especially when precut papillotomy is applied; and, second, the occurrence of a very rare complication of pancreatitis late in the course of this disease.

The incidence of post-ERCP pancreatitis is reported as ranging from 1.3% to 6.7% [1]. There are also studies with an incidence of up to 24% [1]. The varying incidence of post-ERCP pancreatitis depends either on the case mix or the criteria used for defining pancreatitis. Pancreatitis and high serum amylase usually occur after difficult procedures in which pancreatography was achieved [2]. Small common bile ducts and precut papillotomy also significantly increase the risk of pancreatitis [2]. An age of less than 59 years, opacification caused by instillation of radiographic contrast in the pancreatic duct and the absence of common bile duct stones appear to be independent predictors of post-ERCP pancreatitis [3]. Pancreatitis occurs in patients with younger median age and more often in women [2]. The complication can also occur without cannulation and opacification of the pancreatic duct.

The precut technique is performed after the failure of multiple cannulation attempts. Although the precut procedure is reported to be safe [4], it can increase the complication rate of the procedure and should be restricted to cases in which endoscopic intervention is mandatory [5]. This is the case in patients with dilatation of the bile ducts, jaundice, cholangitis or itching due to obstructive jaundice. The case described here had all of the signs of stones in the common bile duct with the exception of laboratory abnormalities. Post-ERCP pancreatitis usually has a good prognosis, and most patients can be discharged within 5 days [6].

Pancreatitis can be a serious condition, and systemic complications in particular add to morbidity and mortality. Fat embolism is reported as a very rare complication. Sporadic cases are reported, mostly in older literature. Fat embolism usually occurs at the onset of the pancreatitis [7,8].

Fat embolism is a well-known complication of fractures of long bones and bone surgery. Fat droplets in small vessels may be derived from the bone marrow or from plasma by agglutination of chylomicrons or by infusion of exogenous fat. This can result in vascular occlusion and infarction. Free fatty acids have a direct toxic effect on endothelial cells and pneumocytes, resulting in capillary leakage and loss of surfactant, and the formation of hyaline membranes.

Classical fat embolism is characterised by the triad of respiratory distress, mental disturbances and petechial skin rash occurring 12 to 72 hours after the initial incident responsible for the fat embolism. The pulmonary fat embolism syndrome exists as a spectrum, from embolism of fat without clinical symptomatology to the full-blown syndrome with a mild or even fulminant presentation.

Fat embolism has also been described in cases of pancreatitis, diabetes, lipectomy, lipid hyperalimentation and sickle cell disease. Chylomicron and very low-density lipoprotein (VLDL) have been shown to develop calcium-dependent agglutination by C-reactive protein in acute pancreatitis [7]. Fat embolism in the course of acute pancreatitis has been described previously [8]. The condition can also occur in the eye, resulting in temporary loss of vision [9,10].

Chest radiography may show a snowstorm pattern in 30% to 60% of patients. Our patient only had pleural effusion on previous X-ray examination of the thorax, and there was no opportunity for repeated radiographic examination of the lungs. This may be an indication of the acute onset of massive lethal fat embolism.

Cerebral infarction due to fat embolism in the course of traumatic pancreatitis more than 2 weeks after the accident has been described previously [11]. Our patient developed acute massive pulmonary fat embolism more than 2 weeks after the onset of pancreatitis. This complication was unusual given the fact that the patient had improved remarkably and was almost ready for discharge. The contrast-enhanced CT scan revealed no signs of pancreatic necrosis. However, the autopsy clearly showed necrosis in the pancreas, although not in the head of the pancreas. It is reasonable to assume that this necrosis triggered the fat embolism.

## Conclusion

This case demonstrates that fat embolism can occur after the remission of pancreatitis and shows that pancreatic necrosis may be overlooked on contrast-enhanced CT scanning.

## Abbreviations

ALAT: alanine aminotransferase; ASAT: aspartate aminotransferase; CT: computed tomography; ERCP: endoscopic retrograde cholangiopancreaticography; VLDL: very low-density lipoprotein.

## Competing interests

The authors declare that they have no competing interests.

## Consent

Written informed consent could not be obtained in this case since the patient's next-of-kin were untraceable. We believe this case report contains a worthwhile clinical lesson which could not be as effectively made in any other way. We expect the patient's next-of-kin not to object to the publication since every effort has been made so the patient remains anonymous.

## Authors' contributions

BK was the attending physician and RL was the consultant gastroenterologist. Both authors have read and approved the final version of the manuscript.
